# NAC1 Maintains Root Meristem Activity by Repressing the Transcription of *E2Fa* in *Arabidopsis*

**DOI:** 10.3390/ijms232012258

**Published:** 2022-10-14

**Authors:** Chuantian Xie, Zhaojun Ding

**Affiliations:** The Key Laboratory of Plant Development and Environmental Adaptation Biology, Ministry of Education, College of Life Sciences, Shandong University, Qingdao 266237, China

**Keywords:** root, meristem, NAC1, E2Fa, endoreduplication

## Abstract

Root meristem is a reserve of undifferentiated cells which guide root development. To maintain root meristem identity and therefore continuous root growth, the rate of cell differentiation must coordinate with the rate of generation of new cells. The *E2 promoter-binding factor a* (*E2Fa*) has been shown to regulate root growth through controlling G1/S cell cycle transitions in *Arabidopsis thaliana*. Here, we found that NAC1, a member of the NAM/ATAF/CUC family of transcription factors, regulated root growth by directly repressing the transcription of *E2Fa*. Loss of *NAC1* triggers an up-regulation of the *E2Fa* expression and causes a reduced meristem size and short-root phenotype, which are largely rescued by mutation of *E2Fa*. Further analysis showed that NAC1 was shown to regulate root meristem by controlling endopolyploidy levels in an E2Fa-dependent manner. This study provides evidence to show that NAC1 maintains root meristem size and root growth by directly repressing the transcription of *E2Fa* in *Arabidopsis*.

## 1. Introduction

Plant meristem is a reserve of undifferentiated cells which guide postembryonic development [1,2,3,4]. In the root meristem of *Arabidopsis*, all root tissues originate from a stem cell niche which includes a small group of slowly dividing cells, quiescent center (QC), and the surrounding stem cells [5]. Stem cell daughters undergo a certain number of cell divisions until they reach the transition zone where divisions cease and cells start to differentiate [5,6]. To maintain root meristem and therefore continuous root growth, the rate of cell differentiation must coordinate with the rate of generation of new cells [1]. A delayed or accelerated transition to elongation causes either an increase or a decrease in root meristem, respectively [1,2].

The root meristem is located at the distal part of the growing root and continuously generates new cells [6]. Two sets of transcriptional routes have been shown to play a key role in the establishment and maintenance of root meristem activity through specifying the QC and stem cell identity [1]. One is the plant hormone auxin, which is transduced through AP2 transcription factors PLETHORAs (PLTs) to control root stem cell niche identity and root meristem activity [1,7]. The other one involves the GRAS family transcription factors SHORTROOT/SCARECROW (SHR/SCR) and their interacting proteins [8,9,10,11]. Mutating each of the key regulators causes the loss of the root QC and stem cell identity, eventually affecting root growth and development [1,12,13]. In addition, other plant hormones such as cytokinin (CK), gibberellin (GA), and brassinosteroids (BR) also play important roles in the maintenance of root meristem identity [14,15,16].

Eukaryotic E2 promoter-binding factors (E2Fs) are transcription factors that are major regulators of cell division, DNA repair, and cell differentiation [17,18]. Among the six E2F transcription factors (E2Fa, E2Fb, E2Fc, EDL1/E2Fe, EDL2/E2Fd, EDL3/E2Ff) in *Arabidopsis* [19], both E2Fa and E2Fb are transcriptional activators, while E2Fc acts as a repressor of the cell cycle [17,20,21]. The other three E2F proteins, repressing E2F-regulated reporter genes, are also functional repressors [18,22]. The E2Fa plays an important role in switching cell division to endoreduplication [23]. Overexpression of both *E2Fa* and its partner *DIMERIZATION PARTNER* (*DPa*) in *Arabidopsis* can stimulate cell proliferation and induce extra rounds of DNA replication, which result cell endoreduplication [17]. The E2Fa/DPa complex, controlling the expression of essential genes during the G1/S transition, can be dissociated by SUMOylation E3 METHYL METHANESULFONATE SENSITIVITY GENE21 (AtMMS21) [24]. In particular, the phenotype of defective root development in *35s::E2Fa-DPa* transgenic seedlings is completely recovered when *AtMMS21* is overexpressed [24]. Additionally, rapamycin (TOR) kinase is essential for the maintenance of mitotic activity by directly phosphorylating E2Fa and E2Fb in both the shoot and root apexes [25,26]. All these results suggest that E2Fa plays an important role in root growth and development. However, the underlying molecular mechanism regarding how E2Fa is finely regulated at the transcriptional level to ensure root meristem identity and root growth is not well understood.

NAC (NAM, ATAF1/2, CUC) domain proteins are unique to plants and comprise large gene families [27]. There are around 105 NAC members which are involved in various aspects of plant development [27,28]. NAC transcription factors are characterized by a highly conserved NAM DNA-binding domain in the N-terminal region, accompanied by diverse C-terminal domains [29]. During lateral root initiation, NAC1 acts as a transcription activator to mediate auxin signaling, and microRNA (miRNA) 164 guides the cleavage of endogenous *NAC1* mRNA in an auxin-dependent manner [30,31,32]. Furthermore, the NAC1 proteins are ubiquitinated and degraded by the 26S proteasome in a SINAT5 E3 controlled manner [31]. Novel functions are described in that *NAC1* is induced in response to wounding and functions in promotion of root rip emergence [33]. However, the mechanistic studies of NAC1 on the functions of plant root growth are still unknown.

In this study, we found that the NAC domain protein NAC1 plays an important role in root growth. NAC1 maintains root meristem size and therefore root growth through directly repressing the transcription of *E2Fa*. The stunted root growth and reduced root meristem size, which result from the decreased cell numbers in root meristem, were largely rescued by mutation of E2Fa in the *nac1* mutant. This study not only expands our knowledge about the biological roles of NAC1, but it also improves our understanding of how the cell cycle regulator E2Fa is delicately regulated by NAC1 to maintain root meristem and root growth.

## 2. Results

### 2.1. NAC1 Is Required for Primary Root Growth

**Figure 1:**
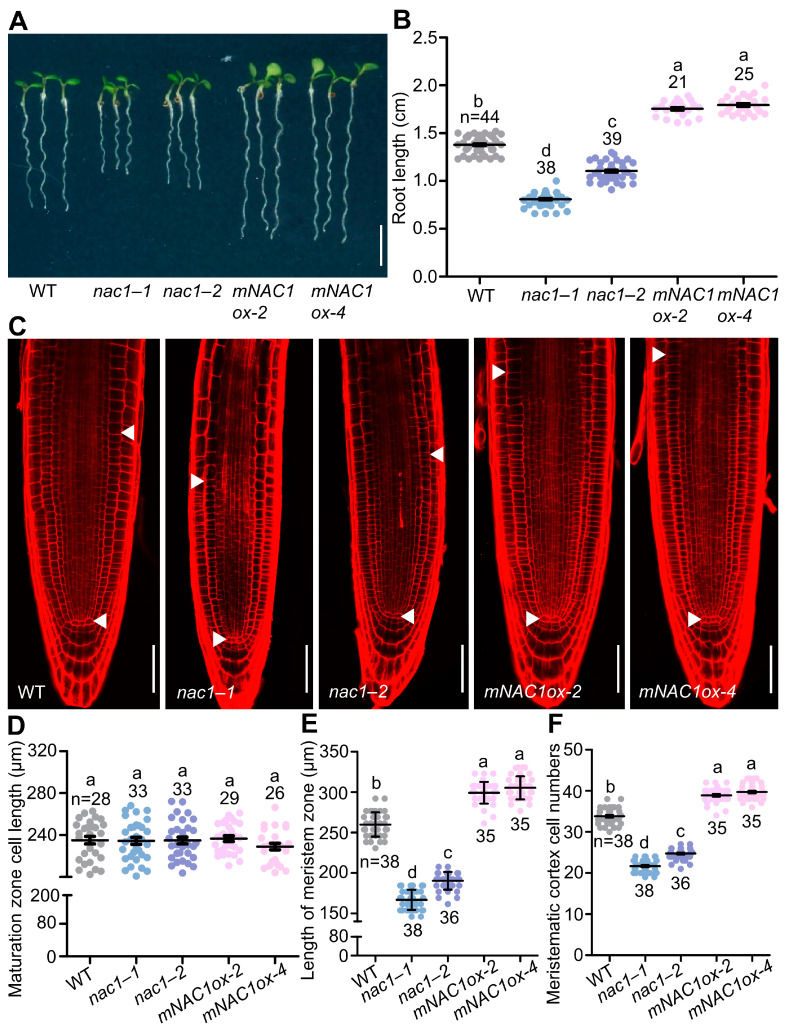
NAC1 is required for primary root growth. (**A**) The root growth phenotypes of WT, *nac1*–*1*, *nac1*–*2*, and two independent overexpression transgenic lines of *mNAC1ox-2* or *mNAC1ox-4* grown on 1/2 MS medium. The seedlings were photographed at 5 days post-germination (dpg). Scale bar represents 0.5 cm. (**B**) Measurements of primary root length of WT, *nac1–1*, *nac1–2*, and two independent overexpression transgenic lines as shown in (**A**). (**C**) Confocal images of root tips of WT, *nac1–1*, *nac1–2*, and two independent overexpression transgenic lines as shown in (**A**). The seedlings were used at 5 dpg for imaging. White arrows indicate the boundary between root meristem and transition zones. White arrows below indicate the quiescent center (QC). Scale bars represent 50 μm. (**D**) Root cortical cell length in the maturation zone of 5 dpg seedlings as shown in (**C**). (**E**) Length of meristem zone of 5 dpg seedlings as shown in (**C**). (**F**) Cell numbers in the proliferation domain of 5 dpg seedlings as shown in (**C**). Data information: in (**B**,**D**–**F**), data represent mean ± SD, n denotes the total number of scored samples. Individual values (black dots) are shown. Different lowercase letters indicate significant differences by one-way ANOVA followed by Tukey’s multiple comparison test (*p* < 0.05).

In contrast to the other members of the family whose expression is restricted to shoot meristem and flower (*NAM*, *CUC2*, *NAP*) or to vascular tissues (*CmNACP*), *NAC1* is one *NAC* family member which is expressed in the root [32]. Although some expression can be detected in leaf primordia, wounding in the leaf explant, or in expanding cotyledons, the highest NAC1 expression is restricted to the lateral root initiation regions and root meristem [32,33]. The role of NAC1 in lateral root development has been highlighted by transducing auxin signals downstream of the F-box protein TIR1 [32], and whether NAC1 also plays a role in primary root growth was unknown. To test this hypothesis, two T-DNA insertion mutants, *nac1–1* and *nac1–2*, were examined and showed defective root growth (Figure 1A). Considering that microRNAs (miRNAs) guised the cleavage of endogenous and transgenic *NAC1* mRNA, we also generated overexpression transgenic plants expressing a cleavage-resistant form of *NAC1* mRNA (*mNAC1*) according to the previous report [30].

**Figure 2:**
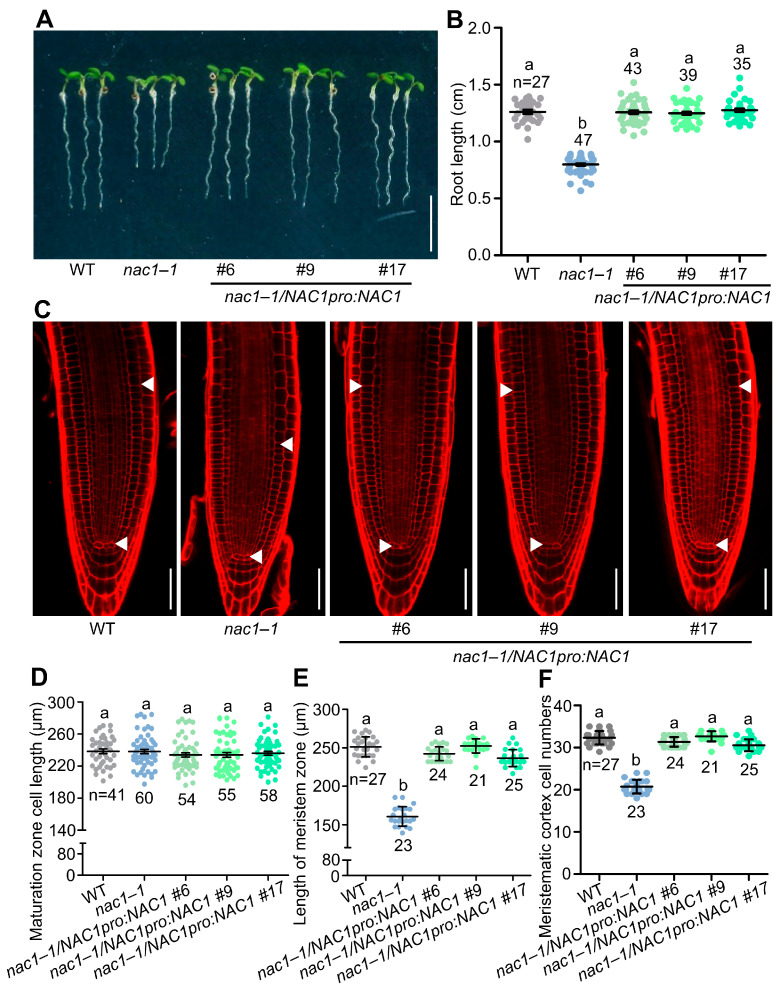
*NAC1pro:NAC1* rescues the short-root-length phenotype in *nac1*–*1*. (**A**) The root growth phenotypes of WT, *nac1*–*1*, and three biological translational transgenic lines of *NAC1pro:NAC1* in *nac1*–*1* mutant background grown on 1/2 MS medium. The seedlings were photographed at 5 dpg. Scale bar represents 0.5 cm. (**B**) Measurements of primary root length of WT, *nac1*–*1*, and three biological translational transgenic lines of *NAC1pro:NAC1* in *nac1*–*1* mutant background as shown in (**A**). (**C**) Confocal images of root tips of WT, *nac1*–*1*, and three biological translational transgenic lines of *NAC1pro:NAC1* in *nac1*–*1* mutant background as shown in (**A**). The seedlings were used at 5 dpg for imaging. White arrows indicate the boundary between root meristem and transition zones. White arrows below indicate the QC. Scale bars represent 50 μm. (**D**) Root cortical cell length in the maturation zone of 5 dpg seedlings as shown in (**C**). (**E**) Length of meristem zone of 5 dpg seedlings as shown in (**C**). (**F**) Cell numbers in the proliferation domain of 5 dpg seedlings as shown in (**C**). Data information: in (**B**,**D**–**F**) data represent mean ± SD, n denotes the total number of scored samples. Individual values (black dots) are shown. Different lowercase letters indicate significant differences by one-way ANOVA followed by Tukey’s multiple comparison test (*p* < 0.05).

Compared to wild-type plants, both *nac1* mutants and *nac1–1/nac1–2* F1 generation mutants had shorter roots, whereas the *mNAC1* overexpression lines displayed longer roots (Figure 1A,B and Figure S1A,B). Similarly, the size of the root apical meristem and meristem cell number were reduced in the *nac1*, *nac1*–*1/nac1*–*2* F1 generation mutants and increased in the *mNAC1* overexpressing lines, whereas the cortical cell length in the root maturation zone was unaltered compared to the wild type (Figure 1C–F and Figure S1C–F). Likewise, root length, root apical meristem size, and meristem cell number were completely or largely restored in *nac1*–*1/NAC1pro:NAC1* compared to *nac1*–*1* (Figure 2A–F), confirming that these phenotypes were caused by a mutation in *NAC1*.

### 2.2. NAC1 Inhibits the Transcription of the Cell Cycle Regulator E2Fa

**Figure 3:**
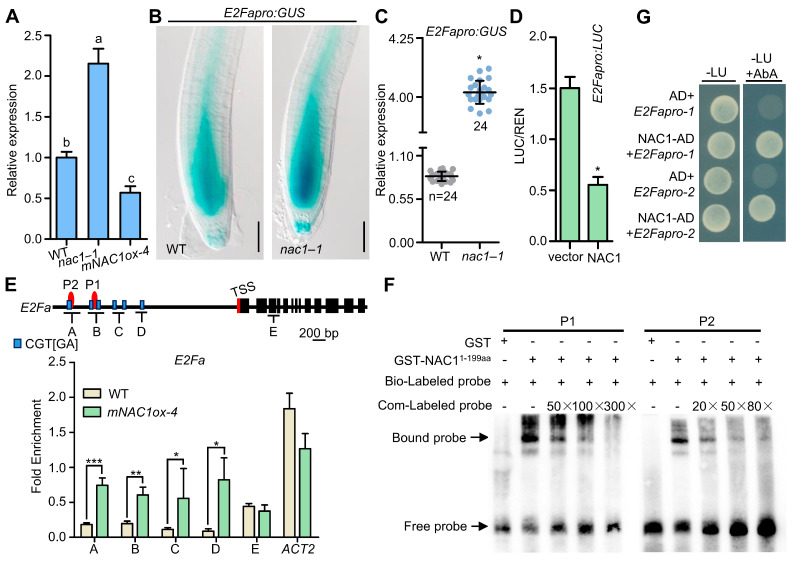
NAC1 directly binds to *E2Fa* promoter and represses its expression. (**A**) RT-qPCR analysis of *E2Fa* expression in WT, *nac1*–*1*, and *mNAC1ox*-*4.* Total RNA was extracted from primary root of 5 dpg seedlings. The expression level in seedlings was defined as “1”. Three biological replicates with three technical replicates for each biological replicate were performed with similar results. Data represent mean ± SE of three biological replicates. Different letters above bars indicate a significant difference (one-way ANOVA, Tukey’ multiple comparisons test, *p* < 0.05). (**B**) Expression of the *E2Fapro:GUS* in WT, *nac1*–*1* roots. *E2Fapro:GUS* crossed with *nac1*–*1* seedling. The seedlings were photographed at 5 dpg. Scale bar represents 50 μm. (**C**) Statistical analysis of *E2Fapro:GUS* expression in (**B**). Data represent mean ± SD, n denotes the total number of scored samples. Individual values (black dots) are shown (Student’s *t* test, * *p* < 0.05). (**D**) NAC1 transrepresses the *E2Fa* promoter in Arabidopsis leaf protoplasts. The effector (*35s::NAC1*) and reporter (*E2Fapro:LUC*) constructs. The empty vector *pBI221* was used as a negative control. Three biological replicates with three technical replicates for each biological replicate were performed with similar results. Data represent mean ± SE of three biological replicates. * means differ significantly (*p* < 0.05) from the negative control. (**E**) Schematic diagram of the *E2Fa* and PCR amplicons (indicated as letters A–E) used for ChIP-qPCR. TSS, transcription start site. ChIP-qPCR results show the enrichment of NAC1 on the chromatin of *E2Fa*. Sonicated chromatins from 5 dpg seedlings (*35s::MYC-mNAC1*) were precipitated with anti-Myc antibodies. The precipitated DNA was used as a template for qPCR analysis, with primers targeting different regions of the *E2Fa* as shown in (**E**). Three biological replicates with three technical replicates for each biological replicate were performed with similar results. Data represent mean ± SE of three biological replicates (*** *p* < 0.001, ** *p* < 0.01, * *p* < 0.05, Student’s *t* test). (**F**) Electrophoretic mobility shift assay (EMSA) shows that NAC1 (1–199aa) binds the putative motif in the *E2Fa* promoter. The biotin-labeled probes (P1 and P2) are indicated in (**E**). Unlabeled probes were used in the competition assay. (**G**) Yeast one-hybrid binding assay involving NAC1 and *E2Fa* promoters. The yeast transformants were dropped onto SD-L-U (-Leu, -Ura) media. Aureobasidin A (AbA) concentration with 290 ng/mL. *E2Fapro-1* spans from −1211 to −647 bp; *E2Fapro-2* spans from −646 to −1 bp.

Considering the essential role of the *E2Fa* gene in controlling cell division and differentiation during the G1/S cell cycle [17,24,34], we first examined the expression of *E2Fa* by RT-qPCR since meristem cell numbers were decreased in the *nac1* mutant (Figure 3A). We observed a higher transcript level of *E2Fa* in *nac1*–*1*, and a decrease in *mNAC1ox-4* overexpression lines compared to wild type (Figure 3A). Consistently, the expression of *E2Fapro:GUS*, which is highly expressed in the meristem of wild-type roots, was dramatically accumulated in *nac1*–*1* roots (Figure 3B,C). Furthermore, to examine whether the NAC1 affects the transcriptional activity of *E2Fa* in plants, we then used a transient transformation *LUC* reporter gene (*E2Fapro:LUC*) and performed transient expression assays in *Arabidopsis* leaf mesophyll protoplasts. The results showed that the *LUC* expression of the *E2Fa* promoter was specifically repressed by NAC1 compared with control (Figure 3D). Taken together, these results suggest that NAC1 inhibits the transcriptional activity of *E2Fa.*

### 2.3. NAC1 Directly Binds to E2Fa Promoter

To further investigate whether NAC1 directly binds to the *E2Fa* promoter in vivo, we performed a chromatin immunoprecipitation (ChIP) followed by a quantitative real-time PCR assay (RT-qPCR) using *NAC1* overexpression seedlings (*mNAC1ox-4*). It was previously reported that the DNA-binding specificity of NAC proteins contain the core CGT[GA] [29]. The global genomic DNA-binding site analysis revealed that the *E2Fa* promoter contains the predicated transcriptional sites of NAC proteins which marked A–D and E negative controls (Figure 3E). Compared with the wild-type seedlings, the A–D but not E fragments of the *E2Fa* promoter were highly enriched in the *mNAC1ox-4* seedlings, indicating that the NAC1 proteins were associated with the genomic regions of the *E2Fa* promoter in vivo (Figure 3E). Furthermore, the electrophoretic mobility shift assays (EMSA) showed that NAC1 bound to the *E2Fa* ChIP-positive fragment (P1 and P2) specifically in vitro, and this interaction could be abolished by adding specific competitor probes (Figure 3F). Consistently, using yeast one-hybrid assays, we observed the interaction between NAC1 and the *E2Fa* promoter in yeast cells, suggesting that NAC1 directly binds to the *E2Fa* promoter (Figure 3G).

Considering that three *Arabidopsis* E2F proteins E2Fa, E2Fb, and E2Fc, but not other E2F factors, have been shown to interact with the retinoblastoma-related (RBR) proteins and play the same roles in G1/S transition [19,35,36], we also studied the relationship between NAC1 and *E2Fb* or *E2Fc*. Similarly, we performed transient expression assays in *Arabidopsis* leaf protoplasts and yeast one-hybrid assays to text whether NAC1 regulated *E2Fb* or *E2Fc* expression (Figure S2A–D). The results showed that NAC1 neither regulates the expression of *E2Fb* nor *E2Fc*, nor binds to their promoter directly in yeast one-hybrid assays, indicating NAC1 specifically inhibits *E2Fa* expression during root development.

### 2.4. E2Fa Acts Downstream of NAC1 to Control Primary Root Growth

**Figure 4:**
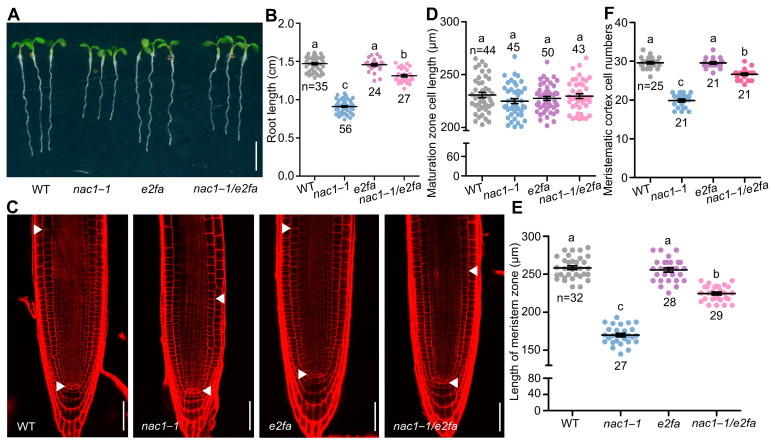
E2Fa acts downstream of NAC1 to control root growth. (**A**) The root growth phenotypes of WT, *nac1–1*, *e2fa*, and *nac1–1/e2fa* grown on 1/2 MS medium. The seedlings were photographed at 5 dpg. Scale bar represents 0.5 cm. (**B**) Measurements of primary root length of WT, *nac1–1*, *e2fa*, and *nac1–1/e2fa* as shown in (**A**). (**C**) Confocal images of root tips of WT, *nac1–1*, *e2fa*, and *nac1–1/e2fa* as shown in (**A**). The seedlings were used at 5 dpg for imaging. White arrows indicate the boundary between root meristem and transition zones. White arrows below indicate the QC. Scale bars represent 50 μm. (**D**) Root cortical cell length in the maturation zone of 5 dpg seedlings as shown in (**C**). (**E**) Length of meristem zone of 5 dpg seedlings as shown in (**C**). (**F**) Cell numbers in the proliferation domain of 5 dpg seedlings as shown in (**C**). Data information: in (**B**,**D**–**F**), data represent mean ± SD, n denotes the total number of scored samples. Individual values (black dots) are shown. Different lowercase letters indicate significant differences by one-way ANOVA followed by Tukey’s multiple comparison test (*p* < 0.05).

**Figure 5:**
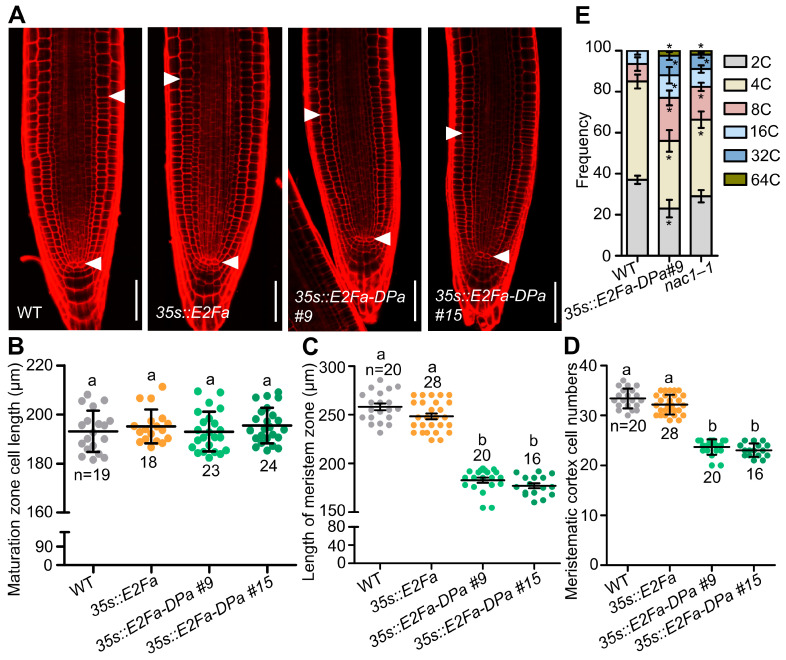
Overexpression *E2Fa-Dpa* complex affects root length by promoting endoreduplication. (**A**) Confocal images of root tips of WT, *35s::E2Fa*, and two independent overexpression transgenic lines of *E2Fa-DPa*. The seedlings were used at 5 dpg for imaging. White arrows indicate the boundary between root meristem and transition zones. Scale bars represent 50 μm. (**B**) Root cortical cell length in the maturation zone of 5 dpg seedlings as shown in (**A**). (**C**) Length of meristem zone of 5 dpg seedlings as shown in (**A**). (**D**) Cell numbers in the proliferation domain of 5 dpg seedlings as shown in (**A**). (**E**) Distribution of nuclear ploidy in the root meristem cells of WT, *35s::E2Fa-DPa #9*, and *nac1–1*, using flow cytometry at 4 dpg. Two independent experiments were performed, and representative results are presented (Student’s *t* test, * *p* < 0.05). Data information: in (**B**–**D**), data represent mean ± SD, n denotes the total number of scored samples. Individual values (black dots) are shown. Different lowercase letters indicate significant differences by one-way ANOVA followed by Tukey’s multiple comparison test (*p* < 0.05).

To further investigate the role of NAC1-mediated inhibition of *E2Fa* in cell division and root growth, we crossed an *e2fa* mutant with the *nac1–1* mutant. We found that several phenotypes of the *nac1–1* mutant were largely recovered in the *nac1–1/e2fa* double mutant, including the shorter root, shorter root apical meristem, and reduced meristem cell number (Figure 4A–F). Significantly, co-overexpression *E2Fa* and *DPa* lines displayed a shorter root meristem and reduced root length compared with the WT [24] (Figure 5A–D). We also examined root meristem cell numbers in co-overexpression *E2Fa* and *DPa* lines. As shown in Figure 5A–D, root meristem cell numbers were reduced in the *35s::E2Fa-DPa* seedlings but not in the *E2Fa* overexpressing lines, implying that the reduced root meristem size and root growth resulted from the decreased root meristem cell numbers in *nac1–1.* Taken together, these results indicated that *E2Fa* acts as a downstream signaling component of NAC1 to maintain root meristem size and root growth.

The previous study showed that E2Fa-DPa was a key regulator of the endocycle [17]. To further study if the elevated *E2Fa* expression caused root growth arrest and might be a result of the regulation of endoreduplication in the *nac1–1* mutant, we performed flow cytometric analysis in the WT, *35s::E2Fa-DPa*, and *nac1–1* plants. The results showed that, similar to the *35s::E2Fa-Dpa* line, the 4 dpg seedling roots of *nac1–1* mutants also showed higher endopolyploidy levels compared with the WT (Figure 5E). These findings indicate that endoreduplication was accelerated by the loss-of-function mutations of *NAC1*, which affected cell division and thus reduced meristem cells in the *nac1* mutant.

## 3. Discussion

**Figure 6:**
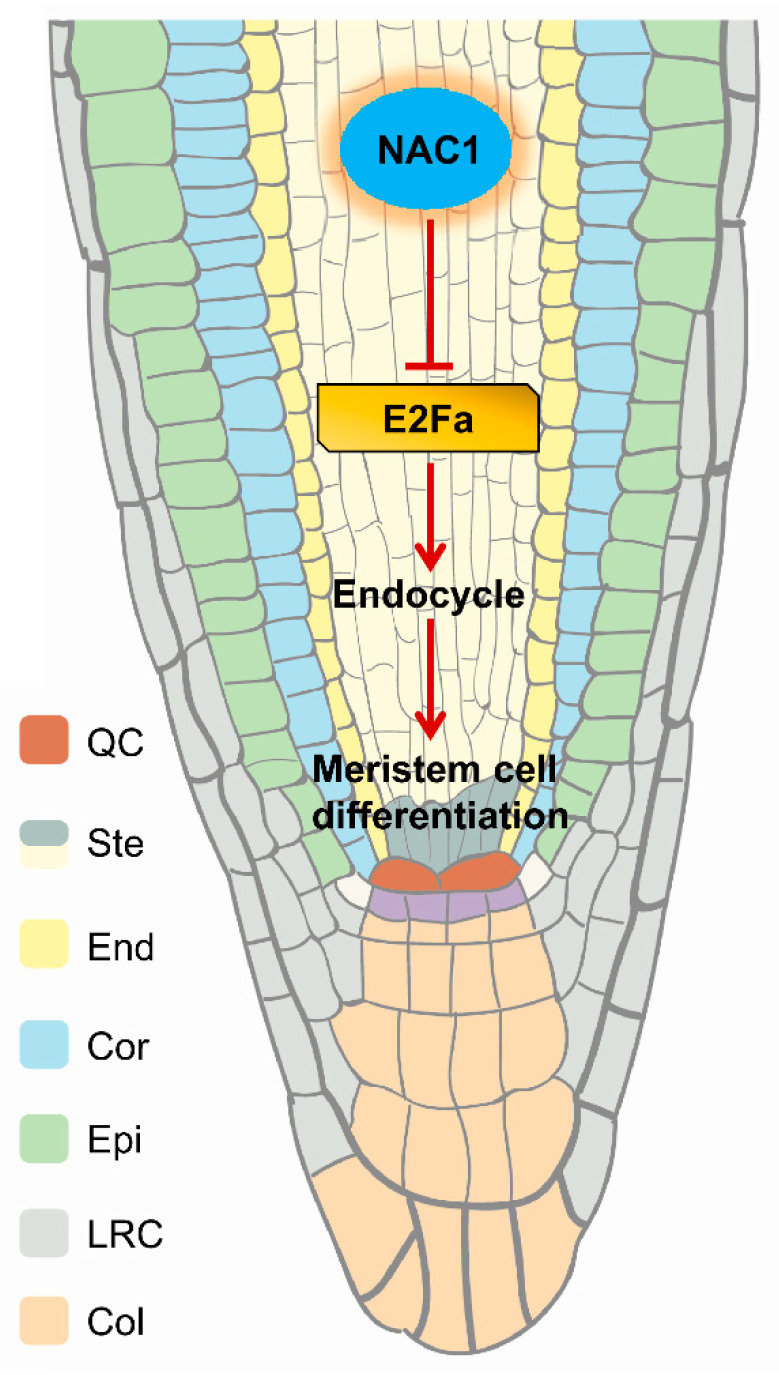
Hypothetical model for the regulation of primary root development by NAC1. NAC1 plays a critical role in primary root growth by directly binding to the promoter and thus repressing the transcription of *E2Fa*. Loss of *NAC1* triggers the up-regulation of *E2Fa* expression, which affects root meristem cell differentiation through the regulation of endocycle.

In *Petunia*, the NAC family gene *NAM* was expressed at the primordial and meristem boundaries, and the mutants failed to develop apical shoots [37]; *Arabidopsis cuc1 cuc2* double mutants displayed highly affected shoot apical meristem development and also exhibited fused cotyledons, sepals, and stamens [38,39]. In contrast to the other members of the NAC family, whose expression is restricted to shoot meristem and flower (*NAM*, *CUC2*, *NAP*) or to vascular tissues (such as, *CmNACP*), as a member of NAC family, NAC1 is highly expressed in roots, and especially, the highest NAC1 expression is restricted to lateral root initiation regions and the root tip (meristem and elongation zone) [32]. Previously data identify NAC1 as a transcription activator in the auxin signaling pathway that regulates genes encoding molecules involved in the specification of lateral root formation [32]. During de novo root organogenesis in *Arabidopsis*, the *NAC1* pathway functioned independently of auxin-mediated explant-specific wounding and root tip emergence [33]. However, the mechanism by which *NAC1* regulates root meristem and root growth is unknown. Here, we provide evidence to show that NAC1 maintains root meristem and root growth through directly repressing *E2Fa* transcription, and loss of *NAC1* triggers up-regulation of the *E2Fa* gene expression, which affects root meristem cell division through the regulation of the endocycle (Figure 6).

E2F/DP transcription factors are well-established targets of the universal CYC-CDK-RBR cascade and key regulators of S-phase genes governing cell cycle progression and DNA replication during postembryonic development, especially in root meristem cell divisions [23,40,41]. On the other hand, the E2Fa-DPa complex not only regulates the mitotic cell cycle progression but also plays a role in the endocycle [17,24]. A previous study showed that E2Fa-DPa was a key regulator of the endocycle [17], and overexpression of *E2Fa* and *DPa* in *Arabidopsis* showed a shorter primary root (Figure 5) [24], which is consistent with the elevated *E2Fa* expression that caused root growth arrest in the *nac1–1* mutant (Figure 4). Furthermore, the transgenic lines overexpressing the *NAC1* were bigger, with larger leaves, thicker stems, and more lateral roots compared with control plants [32]. Considering that endoreduplication is often associated with cell differentiation such as cell growth and organ enlargement, it is reasonable to speculate that the function of NAC1, as a negative regulator of *E2Fa* expression, in maintaining the root meristem size and root growth that are achieved through repressing E2Fa-mediated endoreduplication.

From all the results presented in this study, we put forward an NAC1-E2Fa signaling pathway that controls root meristem growth. However, there are still a couple of unknown questions. For instance, the hormone auxin plays a pivotal role in establishing the root proximodistal axis including meristem, acting as a local signaling factor [3,4], and the *NAC1* gene is induced by auxin [31,32]. Interestingly, the relationship between the NAC1-E2Fa signaling pathway and auxin needs to be clarified in the future. On the other hand, NAC1 acts as a transcription activator in promoting lateral root development [32]. Unexpectedly, we found that NAC1 directly binds to the *E2Fa* promoter to repress its expression; therefore, it should be interesting to find out if NAC1 might regulate specific gene expression through transcriptional activation or repression that is dependent on its partner factors.

## 4. Materials and Methods

### 4.1. Plant Materials and Growth Conditions

In this study, all wild-type, mutant, and transgenic lines are in the Col-0 background; *e2fa* [25], *E2Fapro:GUS* [34], *35s::E2Fa*, and *35s::E2Fa-DPa* [24], as described previously. Mutant seed stocks used in this study are listed in Supplementary Materials. The *nac1–1/e2fa* double mutant was obtained by crossing of *nac1–1* and *e2fa*. The *nac1–1/nac1–2 F1* double mutant was obtained by crossing of *nac1–1* and *nac1–2*. The *nac1–1/E2Fapro:GUS* transgenic line was obtained by crossing of *nac1–1* and *E2Fapro:GUS*.

Plants were sowed on half-strength Murashige and Skoog medium, and then stratified at 4 °C for 2 days in the dark and then transferred to a phytotron set at 22 °C with a 16 h light/8 h dark photoperiod in vertically oriented Petri dishes. Roots were examined at 5 dpg.

### 4.2. PCR, RNA Extraction, and RT-qPCR

The promoter region and coding sequence of *mNAC1* were amplified with Gateway compatible primers (Supplemental Table S1) [30]. The PCR products were first cloned to *pEntry* vector and then recombined with the binary vector *pGWB18* (35s promoter, N-4 × Myc) to generate the *35s::Myc-mNAC1* (*mNAC1ox*) construct. For the *NAC1pro:NAC1* construction, the *NAC1* coding region or promoter regions were first cloned to the *pEntry* vector and then recombined with the binary vector *pGWB1* (no promoter, no tag). All the constructs were transformed into *Agrobacterium tumefaciens* strain GV3101, which was used for transformation of *Arabidopsis* plants by the floral dip method. Transgenes were selected based on their resistance to hygromycin. Homozygous T3 transgenic plants were used for further experiments.

For RT-qPCR analysis, total RNA was extracted form 5 dpg roots using the RNeasy Plant Mini Kit (Qiagen, Hilden, Germany), and cDNA was prepared form 1 μg of total RNA with Hiscript III Reverse Transcriptase (Vazyme) [42,43]. The expression levels of target genes were normalized against *ACT2.* Primers are listed in the Supplementary Materials.

### 4.3. Expression Analysis and Microscopy

For histochemical β-glucuronidase [44] staining, seedlings were infiltrated with 100 mM sodium phosphate buffer (pH 7.2), 0.1% Triton X-100, 2 mM potassium fericyanide and potassium ferrocyanide, 10 mM EDTA, and 2 mM 5-bromo-4-chloro-3-indolyl-β-giucuronide (X-gluc), and incubated at 37 °C for overnight. Samples were cleared in chloral hydrate and visualized with Olympus BX53 microscopy. The process was performed according to Lv et al. [45].

### 4.4. Yeast One-Hybrid Assay

The full-length coding sequences of *NAC1* were amplified with the primers listed in the table in Supplementary Materials and cloned into *pGADT7* vector, and the promoter sequences of *E2Fa*, *E2Fb*, or *E2Fc* were cloned into the *pAbAi* vector. All the constructs used for testing the interactions were transformed into Y1Hgold. The presence of transgenes was confirmed by growth on SD-Ura-Leu plates. Protein interactions were assessed by dropping the yeast transformants on SD-Ura-Leu with Aureobasidin A (AbA) plates. Interactions were observed at 30 °C after 2 days of incubation.

### 4.5. Fluorescence Imaging and Quantification

Root meristems were imaged by a Zeiss LSM 900 laser scanning microscope with a 20× objective. For confocal laser scanning microscopy, root meristems were mounted in 10 μg/mL propidium iodide. The process was performed according to the method described by Tian et al. [44]. In addition, to determine the number of cells belonging to the root meristem, root meristematic cortex cells were counted in a file extending from the QC to the first elongated cell excluded [46]. We quantified root cortical cell length in the maturation zone which has root hairs using 20 to 50 cells from 15 to 20 roots for each genetic background with Image J. Image processing was performed with the LSM image-processing software (Zeiss, Jena, Germany). We determined statistical significance by Student’s *t* test or one-way ANOVA (Tukey’s multiple comparison tests).

### 4.6. EMSA

The GST-NAC1 (1–199 aa) (*pGEX-4T-1*) protein was expressed in *E. coil* BL21 (DE3). We grew BL21 cells at 37 °C in Luria–Bertani (LB) medium in the presence of antibiotics to an OD600 of 0.3 to 0.5. We induced protein accumulation by adding IPTG to a final concentration of 0.3 mM and purified with Glutathione Sepharose 4B (GE Healthcare, Chicago, IL, USA, 17-0756-01) according to the manufacturer’s instructions. EMSA was performed using the LightShift Chemiluminescent EMSA kit (Thermo Scientific, Waltham, MA, USA, 20148) according to the manufacturer’s instructions.

### 4.7. ChIP

ChIP was performed according to the Anne-Valérie Gendrel et al. [47] protocol. Seedlings at 5 dpg were harvested and crossed-linked with 4% formaldehyde under vacuum infiltration, then halted in 2M Gly. Immunoprecipitated chromatin was analyzed by qPCR. Enrichment was calculated as a ratio of bound sequence over input. The expression levels of target genes were normalized against *ACT2.* Primers are listed in Table S1.

### 4.8. Transient Expression

The NAC1 coding sequences were amplified, and the resulting sequences introduced into *pBI221* to place them under the control of the CaMV 35s promoter. The *E2Fa*, *E2Fb*, and *E2Fc* promoter sequences were amplified and introduced into the *pGreenII0800-LUC* vector. Both recombinant plasmids were then transferred into *Arabidopsis* protoplasts. The process was performed according to the method described by Yoo et al. [48]. Primers are listed in the Table S1.

### 4.9. Nuclear Ploidy Analysis

For extraction of nuclei, 4 dpg meristem roots were quickly, finely chopped with a sharp razor blade in 1 mL buffer 1 (100 mM citric acid, 0.5% (*v*/*v*) polysorbate-20, pH 2–3). The process was performed according to the method described by Li et al. [49].

## Data Availability

All data are available in the main text or the Supplementary Materials.

## Supplementary Materials

The following supporting information can be downloaded at: https://www.mdpi.com/article/10.3390/ijms232012258/s1.
