# Supplementary material for: NAC1 Maintains Root Meristem Activity by Repressing the Transcription of E2Fa in Arabidopsis
Source: Int J Mol Sci. 2022 Oct 14;23(20):12258. doi: 10.3390/ijms232012258 (PMC9603599; doi:10.3390/ijms232012258)
Supplement: Supplementary file 1 [file ijms-23-12258-s001.zip › Figures S1 and S2.pdf]

Supplemental Figures

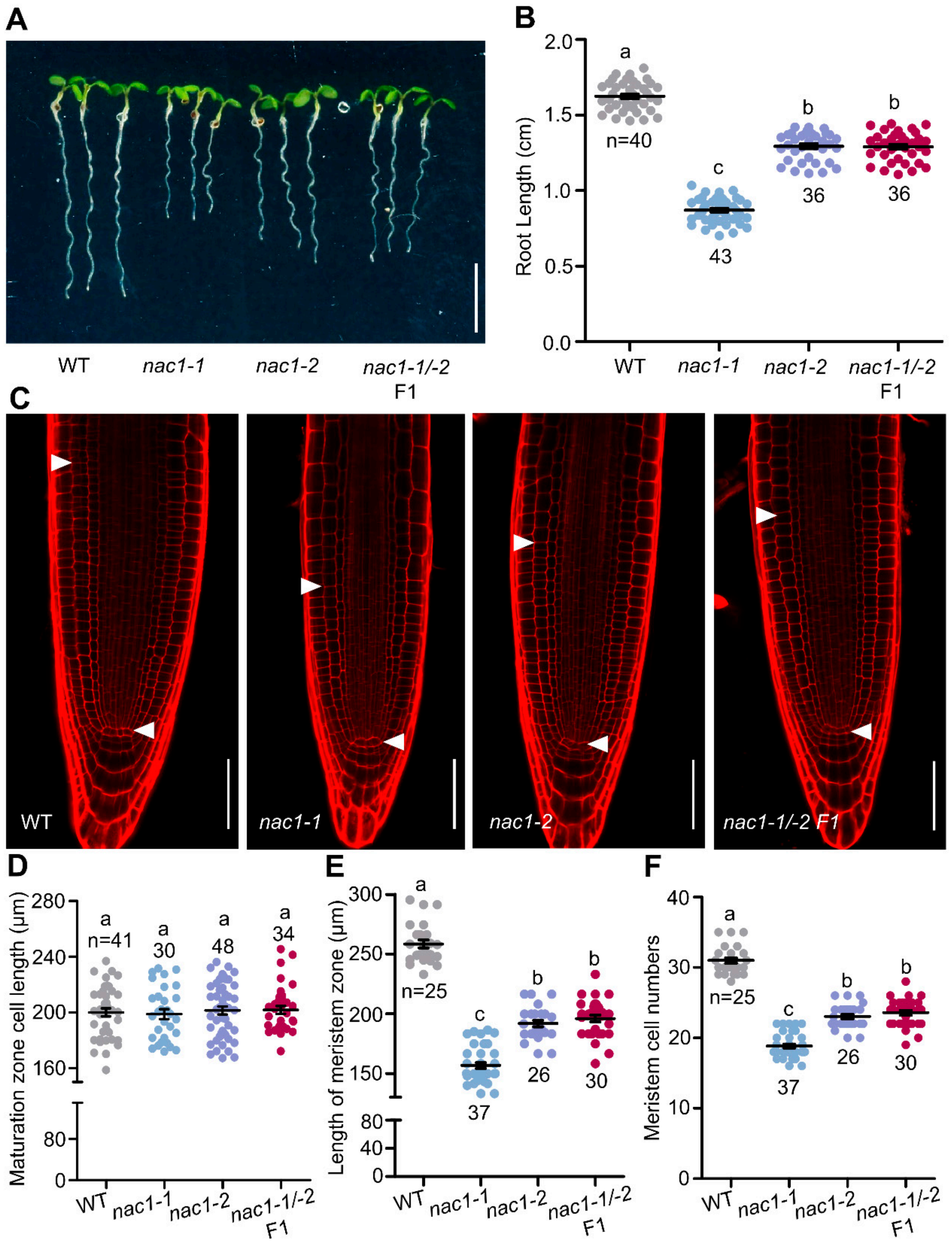

Supplemental Figure S1. Analysis of *nac1-1*/*nac1-2* T-DNA mutants. (**A**) The root growth phenotypes of WT, *nac1-1*, *nac1-2* and the first generation of *nac1-1*/*-2* grown on a 1/2 MS medium. The seedlings were photographed at 5 days postgermination

(dpg). Scale bar represents 0.5 cm. (**B**) Measurements of primary root length of WT, *nac1-1*, *nac1-2* and the first generation of *nac1-1/-2* as shown in (A). (**C**) Confocal images of root tips of WT, *nac1-1*, *nac1-2* the first generation of *nac1-1/-2* as shown in (A). The seedlings were used at 5 dpg for imaging. White arrows indicate the boundary between root meristem and transition zones. White arrows below indicate the QC. Scale bars represent 50 μm. (**D**) Root cortical cell length in the maturation zone of 5 dpg seedlings as shown in (C). (**E**) Length of meristem zone of 5 dpg seedlings as shown in (**C**). (**F**) Cell numbers in the proliferation domain of 5 dpg seedlings as shown in (**C**). Data Information: In (**B**,**D**,**E**,**F**), data represent mean ± SD. *n* denotes the total number of scored samples. Individual values (black dots) are shown. Different lowercase letters indicate significant differences by one-way ANOVA followed by Tukey's multiple comparison test ($P < 0.05$).

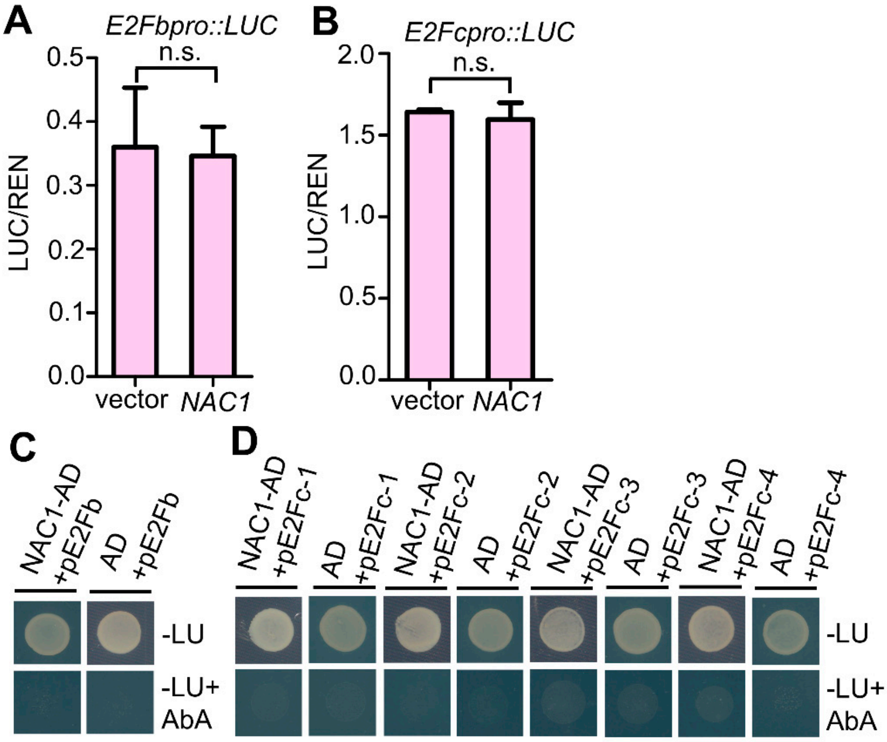

Supplemental Figure S2. NAC1 was not bind the *E2Fb* or *E2Fc* promoter. (**A**,**B**) NAC1 transactivates the *E2Fb* and *E2Fc* promoter in *A. thaliana* leaf protoplasts. The effector (*35s::NAC1*) and reporter (*E2Fbpro::LUC or E2Fcpro::LUC*) constructs. The empty vector pBI221 was used as a negative control. Data represent mean ± SD of

three independent replicates in the experiment (Student's test, n.s., not significant). (**C**,**D**) Yeast one-hybrid binding assay involving NAC1 and *E2Fb* or *E2Fc* promoters. The E2Fbpro spans form -625 to -47 bp were PCR fragment upstream of ATG start site of *E2Fb*; E2Fcpro-1 spans form -4317 to -3574 bp; E2Fcpro-2 spans form -2946 to -2690 bp; pE2Fc-3 spans form -2095 to -1195 bp; pE2Fc-4 spans form -940 to -291 bp were PCR fragment upstream of ATG start site of *E2Fc.* The yeast transformants were dropper onto SD-L-U (-Leu,-Ura) media. AbA concentration with 250 ng/mL.
